# Health care-based voter registration: a new kind of healing

**DOI:** 10.1186/s12245-021-00351-y

**Published:** 2021-04-30

**Authors:** Alister Martin, Ali Raja, Halea Meese

**Affiliations:** 1 MGH Center for Social Justice and Health Equity at Harvard Medical School, Boston, USA; 2 Department of Emergency Medicine, Massachusetts General Hospital, Boston, USA; 3 Department of Family and Community Medicine, University of New Mexico, Boston, USA

## Background

Since the third century AD, physician Yi He has been considered a founder of traditional Chinese medicine. In one of the most famous passages regarding Yi He, the third century BC *Discourses of the States*, he was asked a question that physicians still grapple with over 2000 years later: do healers have a role to play in state affairs? He responded, “The superior physician rescues the state, whereas the inferior one merely attends to the sick.” [1]. His response underscored the ancient Chinese belief that physicians and public servants shared the same purpose. In fact, the terms “physician” and “state minister” were essentially interchangeable in ancient Chinese society. Both terms described healers. Just as physicians were healers of the human body, they were also seen as healers of the body politic.

## Health care-based voter registration

These dual roles of treating both patient and nation animated their practice. In early Chinese stories, the wise state minister and the exemplary physician were often the same person. This too was true during the early years of the USA. Physician statesmen like Benjamin Rush were doctors, signers of the Declaration of Independence, and early slavery abolitionists.

However, the modern roles of physician and statesperson in the USA have become increasingly divergent. One need look no further than voting, the most basic form of political participation, to witness this separation. The limited data available show that physicians are less likely to vote than the general public. In one study, physicians were 9% less likely to vote than the average citizen [2]. The reasons for low rates of voter participation among physicians are not clear. However, it is clear that if physicians want to end health inequities, improve care for the underserved, or help enact laws that dismantle structural racism, they must vote.

There has been some recent progress. Groups like Med Out the Vote (medoutthevote.org) have led voter registration drives within medical schools to prompt medical students and residents to register to vote early in their careers. However, if physicians truly are to be stewards of our nation’s health, they must not only vote, but also help their patients vote too.

The USA has a longstanding history of low voter registration and turnout compared to similar nations. Historically, the demographic groups that are disproportionately more likely to not be registered to vote are those most marginalized by our healthcare system. For example, a disproportionately large share of eligible Americans who were not registered in the 2016 presidential election were people of color and low-income citizens [3]. These are the same demographics who tend to be uninsured, underinsured, or suffer from chronic illnesses shaped by socioeconomic disadvantage. Furthermore, COVID-19 has exacerbated and exposed longstanding healthcare inequalities that disproportionately affect communities of color, the same communities that are less likely to be registered to vote. In other words, the voices missing from the electorate are the same voices that could best inform needed changes to our health system. Therefore, regardless of party affiliation or stance on a particular policy, all physicians should agree that to be pro-voter participation is to be pro-democracy and pro-health.

How can physicians bring more diverse voices into the political process? Simple: ensure that patients are ready to vote. Many physicians are surprised to learn that physician-led voter registration is not a new concept and has been done successfully in various health settings. For example, Massachusetts General Hospital led “MGH Votes,” a voter registration drive sponsored by the Mass General Physician Organization, Physicians for Policy Action, and Nursing and Patient Care Services. Earlier research suggests that patients will welcome this additional service. One intervention conducted in 2012 at two Federally Qualified Health Centers in the Bronx, New York, found that 89% of eligible, unregistered voters were interested in registering at the health center when asked [4]. Both of these examples demonstrate that physicians can engage in voter registration initiatives without disrupting clinic workflows, compromising the patient-doctor relationship, or introducing issues around partisanship.

More recently, groups like Vot-ER (vot-er.org) have made it easier for physicians to help their patients to register to vote. Vot-ER, a nonpartisan, nonprofit healthcare-based voter registration project begun at Harvard Medical School, does so using tools called Healthy Democracy Kits [5]. These complementary kits available to all healthcare providers include a “Ready to Vote?” lanyard, a voter registration badge clipped onto a pre-existing hospital ID, and a text message/QR-code that patients use to register to vote on their own phone. These kits offer an optional way for non-urgent patient populations to start the process of voter registration while they wait in healthcare settings like doctors’ offices and hospitals. Between April and November 2020, a total 24,031 voter registration kits were ordered and used by healthcare providers to help their patients become registered voters. These healthcare providers cumulatively helped 46,712 people to vote in the 2020 presidential election via voter registration or by requesting a mail in ballot to vote from home.

Lastly, physicians increasingly work as part of institutions like hospital systems or health centers that serve as critical touch points within underserved communities. These organizations are beginning to embrace their duty to holistically serve these communities as well as promote anti-racist policies and interventions, and a natural extension of this duty is ensuring that patients have a voice in our democratic process. Some wonder whether it is legal for healthcare spaces to conduct voter registration activities. The law is clear; hospitals and health centers are legally allowed to register patients to vote. The 1993 National Voter Registration Act states that hospitals are permitted to conduct nonpartisan voter education and registration activities. This is the same law that gives the Division of Motor Vehicles (DMVs) the legal ability to do the same work. Due to COVID-19, fewer citizens overall registered to vote in 2020 and countless others were not prompted to update their voter registration due to closures of DMVs and other traditional settings where voter registration typically occurs.

With the increasing focus on the implications of healthcare policy, the legality of healthcare based voter registration, and the emergence of successful models at top tier academic medical institutions nationally, voter registration in healthcare settings is becoming increasingly prevalent and is beginning to galvanize into an organized movement. Over 100 organizations, including the American Academy of Pediatrics, the Student National Medical Association, and the National Association of Community Health Centers, partnered with over 300 hospitals to celebrate the inaugural Civic Health Month in August 2020 to advance the concept of healthcare-based voter registration (civichealthmonth.org). These health institutions, associations, and advocacy organizations are united by the desire to make it easier for patients to register to vote in healthcare settings.

Opponents of voter registration in academic healthcare settings may argue that non-profit healthcare institutions should not become involved in the process of promoting civic engagement for fear of crossing a legal boundary by discussing voter registration in a patient care setting. These critics may not know that the US tax code encourages voter registration to occur at nonprofit 501(c)(3) organizations, which applies to the majority of hospitals in the USA, and the abovementioned National Voter Registration Act similarly encourages the same in all for-profit and government-owned healthcare facilities that treat Medicaid or Medicare patient populations. Voter registration activities in healthcare settings do not pose legal or ethical concerns when they are offered as an optional service for patients and done in a non-partisan, non-coercive, and non-interruptive way.

## Conclusions

Reflection on the words of physician He and consideration of the duality of the physician role in ancient Chinese society suggests that modern US physicians must be just as attuned to the healing of the body politic as the patient body. This must begin with physicians being mindful of our democracy’s health and of who can participate in it.

## Data Availability

As cited.

## Abbreviations

DMV: Department of Motor Vehicles
QR: Quick response
MGH: Massachusetts General Hospital
